# Do we care about appetite?—an investigation into the recording of reduced appetite in older hospitalised adults

**DOI:** 10.1007/s41999-024-00978-z

**Published:** 2024-04-24

**Authors:** Anna Rudzińska, Marcin Wojnarski, Barbara Gryglewska, Jerzy Gąsowski, Karolina Piotrowicz

**Affiliations:** 1 Department of Internal Medicine and Gerontology, Faculty of Medicine, Jagiellonian University Medical College, 2 Jakubowskiego St., Building I, 5th Floor, 30-688, Kraków, Poland; 2 Faculty of Medicine, Jagiellonian University Medical College, Kraków, Poland

**Keywords:** Appetite, Appetite assessment, Appetite disorders, Nutritional care, Geriatrics

## Abstract

**Purpose:**

Reduced appetite is a common issue among older adults. However, its formal assessment is rarely undertaken in clinical practice. The aim of the study was to check the frequency of reporting of appetite status in hospitalized older adults and to analyze the terms documented by physicians when reporting reduced appetite.

**Methods:**

A retrospective analysis of electronic medical records of hospitalized patients aged 65 and older was conducted. To determine patients’ appetite status structured appetite assessment or any references related to appetite were considered.

**Results:**

We included 1291 individual patients’ medical records, of which 13.3% contained any reference to appetite. We showed that in our setting, appetite was not assessed according to standardized questionnaires. In addition, appetite status was documented with inconsistent terminology.

**Conclusions:**

Appetite status was rarely noted in electronic medical records. The lack of a structured assessment of reduced appetite in older patients was found.

## Key summary points

**Aim:**

The aim of the study was to check the frequency of reporting of appetite status in hospitalized older adults and to analyze the terms documented by physicians when reporting reduced appetite.

**Findings:**

In our setting appetite was not assessed using standardized questionnaires. Appetite was mentioned in 13.3% of medical notes.

**Message:**

The lack of routinely performed appetite assessment procedures results in low rates of mention of appetite status in electronic medical records.

## Introduction

The term ‘reduced appetite’ covers various aspects of dietary behaviors and food intake (including selection, motivation, and preferences) [1, 2]. Older adults often suffering from reduced appetite, constitute a significant proportion of recipients of nutritional care and support provided in hospital and outpatient settings [3]. Anorexia of aging, a decrease in appetite associated with aging affects up to 25–30% of older adults, with higher percentage among institutionalized persons [4]. In general, older people consume 16–20% fewer calories, report 25–39% less hunger and 37% higher satiety compared to young adults [5].

According to Polish law, each hospitalized person in Poland should be screened for malnutrition, using Subjective Global Assessment or Nutritional Risk Screening (NRS 2002) scales [6, 7]. Although, due to the legally imposed obligation, nutritional status screening has become common practice in most healthcare facilities, the appetite of the hospitalized patient is not routinely assessed. The Polish Society for Parenteral, Enteral Nutrition and Metabolism (POLSPEN) being the part of The European Society for Clinical Nutrition and Metabolism (ESPEN) undertakes many important projects that intend to raise awareness on the topic of malnutrition. Their campaigns, such as presenting hospitals that maintain high standards of clinical nutrition with the title of *Good Clinical Nutrition Practice Hospital—Treatment through Nutrition* (pl. Szpital Dobrej Praktyki Żywieniowej*—*Leczenie przez żywienie), help to raise awareness of the benefits of preventing malnutrition. At the same time, there are no campaigns in Poland to draw attention to reduced appetite as a problem often preceding the onset of malnutrition.

Although reduced appetite is a common issue among older adults, its routine assessment is rarely undertaken in clinical practice and no guidelines on this topic are available for clinicians.

The lack of research on appetite in older adults also makes it difficult to address both the physiological appetite reduction due to the natural ageing process and pathological anorexia, both of which can lead to health complications. Several standardized and validated questionnaires are currently in use for appetite assessment. These include Appetite, Hunger and Sensory Perception Questionnaire (AHSPQ) and its adaptations such as Council on Nutrition Appetite Questionnaire (CNAQ) and Simplified Nutritional Assessment Questionnaire (SNAQ), or the Functional Assessment of Anorexia and Cachexia Therapy (FAACT) [8–12]. However, there is no agreed gold standard and treatment options for reduced appetite among older adults are still limited. With this in mind, we aimed to find out whether, despite the lack of mandatory appetite screening, physicians pay attention to the problem of appetite changes in old age.

In our study, we aimed to check the frequency of reporting of appetite status, and collect the terms documented by physicians when reporting reduced appetite among older hospitalized patients.

## Methods

We performed a retrospective analysis of the medical electronic records of patients aged 65 and older hospitalized in the Department of Internal Medicine and Geriatrics, University Hospital in Krakow, Poland between January 2019 and December 2020. The search was performed from January 2021 to April 2021.

The electronic medical records were searched using Asseco Medical Management Solutions (AMMS), Rzeszów, Poland, which is the system commonly used for hospital services management.

We searched for results of structured appetite assessment understood as use of any standardized questionnaire or a scale for appetite assessment, or, if unavailable, for any references related to appetite. The following sections of the medical records included in the study were checked in detail: medical interview, physical examination, doctors’ notes, discharge summary, discharge medical recommendations. Given that we analysed records from the period when it was mainly doctors who made entries in the electronic medical system, only doctors’ notes were included, as the system used makes it possible to identify who made the entry. During this period (turnover approx. 650 patients aged 65 years and over/year; 40 beds ward), around 50 physicians were able to enter data into the electronic medical system, including junior doctors who rotate between different hospital wards. The records were checked by two reviewers (AR, MW) and any concerns were resolved through discussion with third reviewer (KP). If there was more than one description for an individual patient, they were analyzed separately.

Information on weight loss was checked in those patients for whom mention on appetite status in electronic medical records was found.

As per exclusion criteria, medical notes of patients who were younger than 65 years (n = 382), those whose hospital stay was shorter than 2 days or those who died during the hospitalization (n = 74 patients) were not included in indepth analysis (n = 1291).

The study was approved by the Bioethics Committee of the Jagiellonian University, 1072.6120.284.2020.

## Results

Of 1291 individual patients’ medical records, 172 (13.3%) included information about appetite, of which 163 referred to reduced appetite and 9 to normal appetite. There was no report on excessive appetite. Of the patients with mention on appetite status, the mean age was 81.4 (SD 8.7) with 58.1% aged 80 years or more; 66.9% of the studied group were women.

Of the 172 patients with information on appetite status included in the study, 58 reported experiencing weight loss before hospital admission. Of the 163 patients with reduced appetite, 54 reported experiencing weight loss before hospital admission.

Of the patients with reduced appetite 89.6% were diagnosed with at least one chronic disease and in 68.1% the coexistence of at least 2 chronic conditions was reported. The most common diseases reported for patients with appetite loss were: kidney disease (33.7%), anemia (30.1%), heart failure (27.6%), dementia (20.9%), diabetes (20.9%), psychiatric diseases (16.6%), liver disease (11.1%), osteoporosis (13.5%), gastrointestinal cancers (9.9%), hematologic malignancy (6.1%), other cancer (11.4%), chronic obstructive pulmonary disease (COPD) (6.8%), autoimmune diseases (1.8%). In 23.3% of the patients with appetite loss the presence of bedsores was reported in the medical documentation. Patients with loss of appetite had on average (SD) 7.5 (4.3) (min–max: 0–21) prescribed medications at the hospital admission.

We found out, that none of the mentions concerning appetite status was based on the results of any standardized questionnaire or a scale for appetite assessment.

**Fig. 1:**
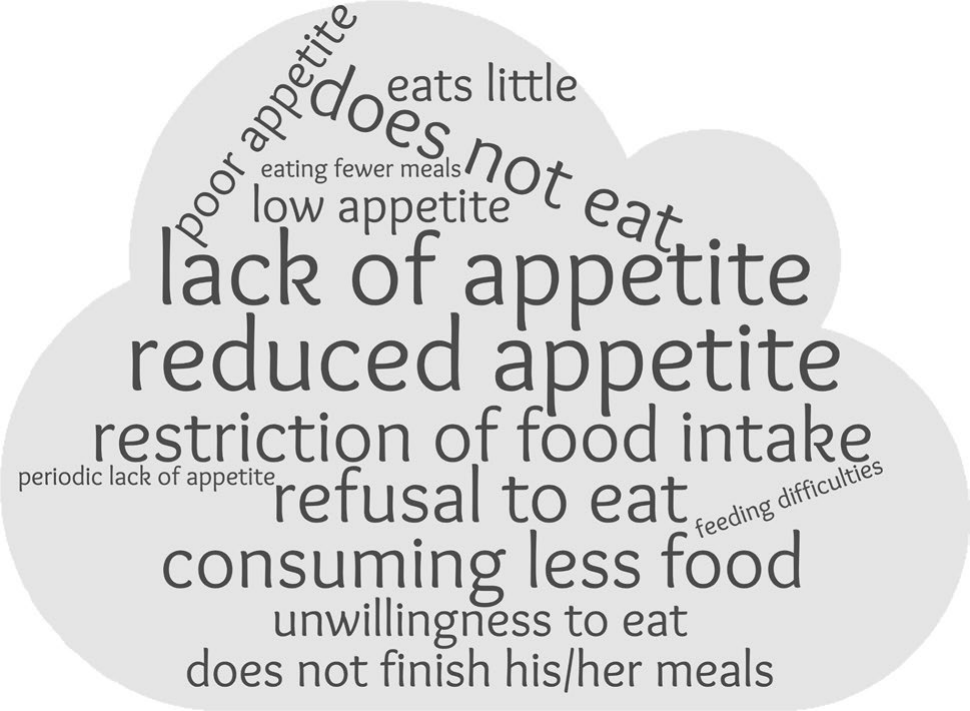
Terms commonly documented by physicians when referring to appetite loss. The wordcloud was created using Wordclouds.com website. The size of a word indicates the frequency of its use in the documentation analysed

The following terms were documented by physicians: lack of appetite (34.4%), reduced appetite (16.6%), does not eat (12.3%), restriction of food intake (7.4%), refusal to eat (6.1%), consuming less food (5.5%), unwillingness to eat (4.9%), low appetite (31.3%), eats little (2.5%), poor appetite (1.8%), experienced problems with food intake (1.8%), does not finish meal (1.8%), disturbed appetite (1.2%), gradual decrease in appetite (0.6%), temporary lack of appetite (0.6%), eating fewer meals (0.6%), feeding difficulties (0.6%) (Fig. 1).

For normal appetite, the mentions were: normal appetite (3.5%), denies loss of appetite (0.6%), improved appetite (0.6%), good appetite (0.6%).

## Discussion

Based on a large sample of electronic medical records, we demonstrated that appetite was rarely reported in hospitalized older adults. When it was present, it was documented with non-standardized terminology and the terms documented to describe it were far from consistent. In the hospital where the study took place, there has been no SOPs or formal procedures for appetite assessment, and the only nutrition-related assessment routinely performed is nutritional risk screening, which does not include questions about patients’ appetite. As there is currently no guideline-based standard for appetite assessment and nomenclature of appetite alterations, and food intake remains unstandardized we might assume that the described problem is not only local. A similar problem was highlighted by Puelle and colleagues for delirium [13]. Together with our results, it stresses the importance of detailed medical notes assessing problems of old age in order to timely recognize these issues and undertake appropriate action.

The lack of an established diagnosis of reduced appetite in hospitalized geriatric patients has important implications for clinical practice. It negatively influences communication between healthcare professionals, both in inpatient and outpatient settings, thus limiting patients’ access to evidence-based nutritional care services.

Despite the high prevalence of reduced appetite due to anorexia of ageing reported in the literature, its occurrence in clinical practice is often overlooked and considered by healthcare professionals as part of the physiological aging process [14, 15]. This false belief is often shared by patients themselves and their caregivers, and results in underreporting of reduced appetite to healthcare practitioners.

In our sample of 1291 medical records of hospitalized patients, only 172 had references to appetite status. The high proportion of patients who might not have received appropriate nutritional counselling to prevent or treat malnutrition demands a change in the paradigm of appetite assessment practices. Having identified the lack of a structured approach to reduced appetite as a problem, it is important to consider what measures can be taken to better identify older patients with this deficit. Although there are some tools available to address the issue of reduced appetite that may be considered helpful such as CNAQ or SNAQ questionnaires, their routine use in the clinical setting may be seen as an unnecessary burden in a high workload situation. Instead, we might propose a multidisciplinary approach. Firstly, a medical interview should include a question about appetite status and changes in body weight. In addition to the standard medical interview, nutritional assessment and counselling should be offered to older adults, especially if the medical interview reveals information about existing malnutrition, unintentional weight loss and reduced appetite. The dietitian may choose to use the tools mentioned above in order to objectify assessment of the patient’s eating behaviour. Moreover, the communication between healthcare professionals should be facilitated to allow for a good exchange of information regarding the amount and quality of intake and the possible need for further steps of nutritional intervention.

It is important to recognise that nutrition is increasingly seen as an integral part of hospital care. Young doctors, whose curricula now include nutrition, have a role in this [16]. The level of education of dietitians is also changing. In Poland, dietitians are now trained at bachelor and master level. However, the profession of dietitian is not yet regulated and the field of study has no defined learning outcomes. As a result, universities are characterised by a high degree of autonomy in the selection of learning outcomes and a wide variety of educational programmes. This is likely to affect the quality of care provided by dietitians to older people, as not all medical universities include appetite-related topics in the curriculum for dietetics students. Dietitians should be able to perform detailed assessment of nutritional status, patients’ food intake and appetite. Given the important role of nutrition in the patient’s treatment process, it should be noted that dietitians should be considered an integral part of the team providing care to the older patient. Somers et al., in a review on clinical approach to the older person with anorexia, suggest an approach that involves collaboration between different health professionals [17]. The authors also draw attention to the watchful waiting period that follows all diagnostic procedures and the reassessment of patients’ nutritional and appetite status after 6–8 weeks. We believe that, as reduction in appetite often precedes the cascade of adverse effects of ageing, providing patients with adequate nutritional care after discharge from hospital where reduced appetite has been diagnosed and their further reassessment can be seen as a preventive measure against falls, sarcopenia and frailty. In addition, as knowledge on geriatric syndromes, including anorexia of aging is considered to be relatively low, the awareness-raising activities about the problems of older people should be undertaken among healthcare professionals [18]. The Geriatric Assessment is considered the gold standard in geriatric care, but in Poland, due to the shortage of geriatric healthcare professionals, the people who would benefit most from this type of complex assessment may not be able to access its benefits. What is more, older adults experiencing the reduced appetite, which is far more subtle and may not be associated with marked changes in external appearance, may not receive the appropriate treatment.

Further research into reduced appetite among the older population is needed to provide evidence-based care for patients experiencing this problem. The ICFSR, in its Task Force Report: Assessment and Management of Appetite Loss in Older Adults, highlights the importance of research into reduced appetite in older age, such as the phenotypes of patients who experience it. According to the ICFSR, the reduced appetite with and without weight loss, the co-occurrence of depression and dementia, and loss of mobility should be studied [19]. According to our research another important direction in research related to appetite in old age is the problem of its reporting and establishing consistent terminology. We suggest that one area for research would be to identify differences in reporting of reduced appetite, as countries differ in terms of eating habits. It can be assumed that older people place different importance on the occurrence of reduced appetite depending on the cultural attribution placed on eating behaviors in different countries. Countries also differ in the amount of money spent on providing care for the aged and the importance of this area of care in the health care system. It remains to be examined whether these differences may be relevant to the reporting of nutrition-related problems among older patients.

Our study had some limitations. Due to fact that the information concerning appetite alterations was placed in various sections in the documentation, as well as using descriptions that often did not allow us to determine the reason for intake restriction, we cannot exclude the possibility of a systematic error. Also, we did not collect data from patient records that were not included in the detailed analysis. As there are no formal requirements to perform appetite screening among older hospitalized adults, reduced appetite could only be recorded in people who formally reported the condition. It is also possible, that reduced appetite was reported but was not adequately documented. Finally, our work was focused on the reduced appetite and we did not investigate the frequency of formal recognition of malnutrition. Exploring the co-occurrence of reduced appetite and malnutrition seems to be an important area for further research.

## Conclusion

Appetite was rarely and inconsistently documented in older adults during hospital stay. Lack of a structured screening and diagnostic approach to older patients presenting with reduced appetite was found.

## Data Availability

The data are not available for deposition in a public repository.
